# Free Vibration Analysis of Spinning Sandwich Annular Plates with Functionally Graded Graphene Nanoplatelet Reinforced Porous Core

**DOI:** 10.3390/ma15041328

**Published:** 2022-02-11

**Authors:** Tianhao Huang, Yu Ma, Tianyu Zhao, Jie Yang, Xin Wang

**Affiliations:** 1 Key Laboratory of Structural Dynamics of Liaoning Province, School of Sciences, Northeastern University, Shenyang 110819, China; tianahao_huang@163.com (T.H.); 2000299@stu.neu.edu.cn (Y.M.); 2 School of Engineering, RMIT University, P.O. Box 71, Bundoora, VIC 3083, Australia; 3 Department of Kinesiology, Shenyang Sport University, Shenyang 110102, China; wangxin@syty.edu.cn

**Keywords:** sandwich annular plate, graphene nanoplatelets, porosity, spinning, free vibration

## Abstract

This paper conducted the free vibration analysis of a sandwich annular thin plate with whirl motion. The upper and lower faces of the annular plate are made of uniform solid metal, while its core is porous foamed metal reinforced by graphene nanoplatelets (GPLs). Both uniform and non-uniform distributions of GPLs and porosity along the direction of plate thickness which leads to a functionally graded (FG) core are taken into account. The effective material properties including Young’s modulus, Poisson’s ratio and mass density are calculated by employing the Halpin–Tsai model and the rule of mixture, respectively. Based on the Kirchhoff plate theory, the differential equations of motion are derived by applying the Lagrange’s equation. Then, the assumed mode method is utilized to obtain free vibration behaviors of the sandwich annular plate. The finite element method is adopted to verify the present model and vibration analysis. The effects of porosity coefficient, porosity distribution, graphene nanoplatelet (GPL) distribution, graphene nanoplatelet (GPL) weight fraction, graphene nanoplatelet length-to-thickness ratio (GPL-LTR), graphene nanoplatelet length-to-width ratio (GPL-LWR), spinning speed, outer radius-to-thickness ratio and inner radius-to-thickness ratio of the plate, are examined in detail.

## 1. Introduction

Spinning disks are widely applied in a rotor machinery, such as aero engines, gas turbines, and so on. The thick disk is commonly adopted in the traditional rotor structures to achieve great structural stiffness and it can be considered as a rigid body in the vibration analysis. To meet the requirements of high spinning speed and light weight, however, thin disks are increasingly used in practical engineering applications. In such cases, the flexibility and the deformation of the disk can no longer be ignored. Theoretically, the thin disk can be modeled as an elastic annular thin plate, whose vibration behaviors have been extensively investigated [1,2,3,4,5].

By employing the finite element method, Pan et al. [6] studied the vibration of rotor bearing-disk system subjected to three forces. Yang et al. [7] developed a thermal stress stiffening method to investigate the vibration behavior of spinning flexible disks. Maretic et al. [8] proposed vibrations of spinning annular plate with two different materials. By adopting the experimental method, Kang et al. [9] studied the vibration characteristics of spinning disk in an air-filled enclosure. The Ritz method is used by Kang et al. [10] to study the free vibration of spinning annular plates with variable thickness. Rao et al. [11] concerned with free vibration behaviors of an annular plate resting on Winkler foundation. Based on Mindlin plate theory, Chen et al. [12] studied the high-frequency vibration performance of an annular plate. Tan et al. [13] deal with the forced and free vibration of a thin annular plate with variable stiffness. Amin et al. [14] investigated the nonlinear vibration behaviors of an FG annular plate. Wang et al. [15] studied the free vibration of an annular plate with different edges.

Due to the spinning effect, the disk is always subjected to aerodynamic loading on its faces. To enhance structural stiffness and reduce weight, a sandwich annular plate structure could be designed in which the upper and lower faces are made of uniform solid metal and the core is porous foamed metal. Because the pores can weaken the structural stiffness, some reinforcements need to be added. GPLs, owing to their superior mechanical properties, are well suited to be the reinforcements. Recently, the vibration behavior of GPL reinforced structures for better mechanical performance has been a topic of extensive research efforts [16,17,18,19,20]. Yang and Zhao et al. [21,22,23,24] carried out extensive research on the free vibration of rotor structures reinforced by GPLs. Based on the modified couple stress theory, Adab et al. [25] studied the free vibration of a spinning sandwich micro-shell. Saidi et al. [26] investigated vibrations of an FG porous GPL reinforced plate subjected to aerodynamical loading. Li et al. [27] studied nonlinear vibrations of a sandwich FG porous GPL reinforced plate resting on Winker–Pasternak elastic foundation. Zhou et al. [28] investigated vibrations of a GPL reinforced porous cylindrical panel under supersonic flow. Gao et al. [29] conducted nonlinear free vibration analysis of a porous plate reinforced with GPLs. Baghlani et al. [30] studied uncertainty propagation in free vibration of an FG porous shell with GPL reinforcement. Anamagh et al. [31] developed a spectral-Chebyshev approach to study vibrations of an FG porous plate reinforced with GPLs. Based on a trigonometric shear deformation theory, Anirudh et al. [32] discussed the vibration behavior of a GPL reinforced FG porous beam.

For a disk with high spinning speed, sandwich structure with a functionally graded graphene nanoplatelet reinforced porous core and stiff faces is an ideal option due to its light weight yet great structural stiffness. To the best of the authors’ knowledge, however, none of the existing studies, including those mentioned above, has discussed the dynamic behaviors of such a spinning disk. This paper aims to fill in this research gap by studying the free vibration of a spinning sandwich annular plate with FG-GPL reinforced porous core. Considering the whirl motion, the annular plate is modeled by the Kirchhoff plate theory. The differential equations of motion and free vibration results are obtained by employing the Lagrange’s equation and assumed mode method, respectively. A comprehensive study is proposed to examine the effects of the material and structural parameters on the natural frequencies of the spinning annular plate. The presented conclusions can effectively aid the design of spinning annular plates with GPL reinforced porous core.

## 2. Theoretical Formulations

### 2.1. Modeling

**Figure 1:**
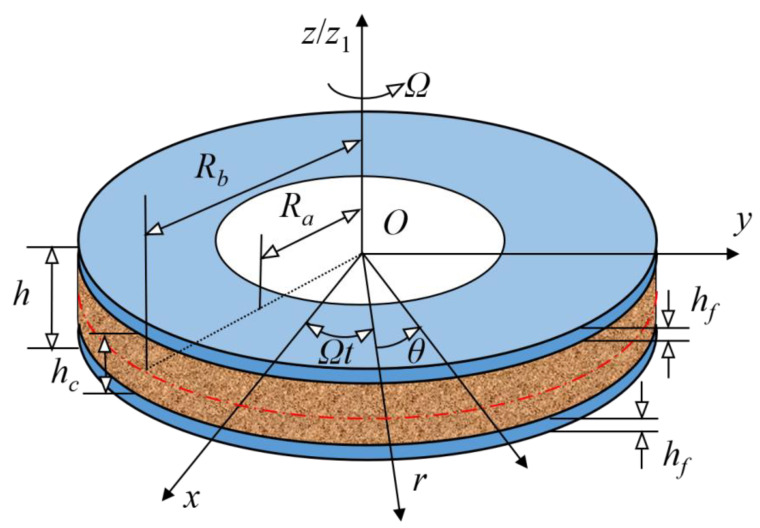
The spinning annular plate model with GPL reinforced porous core and solid faces.

Figure 1 plots the spinning annular plate model with GPL reinforced porous core and solid faces. The inner radius and outer radius of the annular plate are *R_a_* and *R_b_*, respectively. The thickness of the annular plate, the core and the face are *h*, *h_c_ and h_f_,* respectively. To describe the motion and deformation of the spinning annular plate, both the fixed coordinate system (*O*-*xyz*) and polar coordinate system *O*-*rθz*_1_ are established. The annular plate rotates at a constant speed *Ω* along *z*_1_-axis direction.

### 2.2. Material Properties

**Figure 2:**
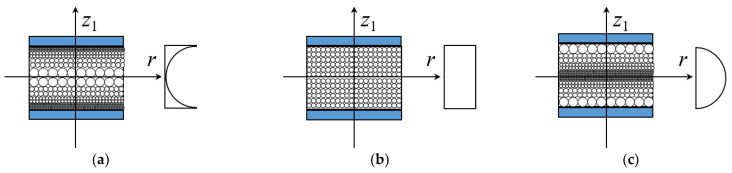
Porosity distribution patterns for the core of the annular plate. (**a**) Pattern X*_P_*; (**b**) Pattern U*_P_*; (**c**) Pattern O*_P_*.

As given in Figure 2, three porosity distributions of the core are considered. Figure 2a plots the positive trigonometric porosity distribution X*_P_*, where more pores are set around the surfaces of the annular plate and less pores are in the middle plane. Based on the open-cell scheme [33], the effective material properties are

(1) $$\mathrm{Porosity}\;\mathrm{Pattern}\;X_{P}\;\left\{ \begin{array}{l} E_{c}\left(z_{1}\right)=E_{c0}\left[1-e_{c0}\cos\left(\pi z_{1}/h_{c}\right)\right]\\ \rho_{c}\left(z_{1}\right)=\rho_{c0}\left[1-e_{cm}\cos\left(\pi z_{1}/h_{c}\right)\right]\\ \mu_{c}\left(z_{1}\right)=\mu_{c0} \end{array}\right.$$

Figure 2c shows the negative trigonometric porosity distribution O*_P_*, where fewer pores are arranged around the surfaces of the annular plate and more pores are in the middle plane. The expressions of material properties are

(2) $$\mathrm{Porosity}\;\mathrm{Pattern}\;O_{P}\;\left\{ \begin{array}{l} E_{c}\left(z_{1}\right)=E_{c0}\left[1-e_{c0}^{*}\left(1-\cos\left(\pi z_{1}/h_{c}\right)\right)\right]\\ \rho_{c}\left(z_{1}\right)=\rho_{c0}\left[1-e_{cm}^{*}\left(1-\cos\left(\pi z_{1}/h_{c}\right)\right)\right]\\ \mu_{c}\left(z_{1}\right)=\mu_{c0} \end{array}\right.$$

Besides, Figure 2b shows the uniform porosity distribution U*_P_*. The material properties are obtained as

(3) $$\mathrm{Porosity}\;\mathrm{Pattern}\;U_{P}\;\left\{ \begin{array}{l} E_{c}\left(z_{1}\right)=E_{c0}\alpha_{c}\\ \rho_{c}\left(z_{1}\right)=\rho_{c0}{\alpha^{\prime }}_{c}\\ \mu_{c}\left(z_{1}\right)=\mu_{c0} \end{array}\right.$$

where *E_c_*, *ρ_c_* and *μ_c_* are the Young’s modulus, mass density and Poisson’s ratio of the core, respectively, while *E_c_*_0_, *ρ_c_*_0_ and *μ_c_*_0_ are the corresponding parameters of the core without pores, respectively; (*e_c_*_0_, *e_c_*_m_) are the porosity coefficient and mass density coefficient of Pattern X*_P_*, while (e^*^_c__0_, e^*^_c__m_) and (*α_c_*, *α’_c_*) are the corresponding parameters of Pattern O*_P_* and U*_P_*, respectively.

Due to the typical mechanical property, the mass density coefficients and porosity coefficients are related by

(4) $$\left\{ \begin{array}{l} 1-e_{cm}\cos\left(\pi z_{1}/h_{c}\right)=\sqrt{1-e_{c0}\cos\left(\pi z_{1}/h_{c}\right)}\\ 1-e_{cm}^{*}\left(1-\cos\left(\pi z_{1}/h_{c}\right)\right)=\sqrt{1-e_{c0}^{*}\left(1-\cos\left(\pi z_{1}/h_{c}\right)\right)}\\ {\alpha^{\prime }}_{c}=\sqrt{\alpha_{c}} \end{array}\right.$$

According to the principle of equal mass, the mass density coefficients of different porosity distribution are determined by

(5) $$\left\{ \begin{array}{l} \int_{0}^{h_{c}/2}\sqrt{1-e_{c0}^{*}\left(1-\cos\left(\pi z_{1}/h_{c}\right)\right)}dz_{1}=\int_{0}^{h_{c}/2}\sqrt{1-e_{c0}\cos\left(\pi z_{1}/h_{c}\right)}dz_{1}\\ \int_{0}^{h_{c}/2}\sqrt{\alpha_{c}}dx_{0}=\int_{0}^{h_{c}/2}\sqrt{1-e_{c0}\cos\left(\pi z_{1}/h_{c}\right)}dz_{1} \end{array}\right.$$

Based on the Halpin–Tsai model [34], *E_c_*_0_ can be given by

(6) $$E_{c0}\left(z_{1}\right)=E_{M}\left[\frac{3}{8}\left(\frac{1+\xi_{lc}\eta_{lc}V_{GPL}}{1-\eta_{lc}V_{GPL}}\right)+\frac{5}{8}\left(\frac{1+\xi_{wc}\eta_{Bc}V_{GPL}}{1-\eta_{Bc}V_{GPL}}\right)\right]$$

(7) $$\eta_{lc}=\frac{E_{GPL}/E_{M}-1}{E_{GPL}/E_{M}+\xi_{lc}},\eta_{Bc}=\frac{E_{GPL}/E_{M}-1}{E_{GPL}/E_{M}+\xi_{wc}}$$

(8) $$\xi_{lc}=2l_{c}/t_{c},\xi_{wc}=2w_{c}/t_{c}$$

in which *E_M_* and *E_GPL_* are Young’s modulus of the foam metal matrix and GPLs, respectively; *l_c_*, *w_c_* and *t_c_* are the length, width and thickness of GPLs, respectively.

In accordance with the rule of mixture, it can be obtained as

(9) $$\left\{ \begin{array}{l} \rho_{c0}\left(z_{1}\right)=V_{GPL}\rho_{GPL}+\left(1-V_{GPL}\right)\rho_{M}\\ \mu_{c0}\left(z_{1}\right)=V_{GPL}\mu_{GPL}+\left(1-V_{GPL}\right)\mu_{M} \end{array}\right.$$

where *ρ_GPL_* and *μ_GPL_* are the mass density and Poisson’s ratio of GPLs, respectively, while the*ρ_M_* and *μ_M_* are the corresponding parameters of the foam metal matrix, respectively.

**Figure 3:**
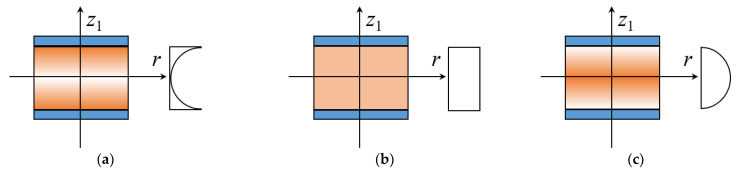
GPL distribution patterns for the core of the annular plate. (**a**) Pattern X*_G_* (**b**) Pattern U*_G_* (**c**) Pattern O*_G_*.

As shown in Figure 3, three GPL distribution patterns of the core are taken into consideration. Figure 3a illustrates the positive trigonometric GPL distribution X*_G_*, where more GPLs are adding around the surfaces of the core and less GPLs are in the middle plane, while Figure 3c gives the opposite GPL distribution O*_G_* and Figure 3b indicate the uniform GPL distribution U*_G_*.

The expression *V_GPL_* of volume fraction of GPLs corresponding to the above three GPL distributions can be expressed as

(10) $$V_{GPL}\left(z_{1}\right)=\left\{ \begin{array}{ll} \lambda_{1}\left[1-\cos\left(\frac{\pi z_{1}}{h_{c}}\right)\right] & {\mathrm{Pattern}\;X}_{G}\;\\ \lambda_{2}\;\mu & {\mathrm{Pattern}\;U}_{G}\\ \lambda_{3}\cos\left(\frac{\pi z_{1}}{h_{c}}\right) & {\mathrm{Pattern}\;O}_{G}\; \end{array}\right.$$

in which (*λ*_1_, *λ*_2_, *λ*_3_) is the volume fraction index. They can be determined by the GPL weight fraction *W_GPL_* in the form of

(11) $$W_{GPL}=\int_{-\frac{h_{c}}{2}}^{\frac{h_{c}}{2}}\left[\rho_{c}\frac{\rho_{GPL}V_{GPL}}{\rho_{GPL}V_{GPL}+\rho_{M}\left(1-V_{GPL}\right)}\right]dz_{1}/\int_{-\frac{h_{c}}{2}}^{\frac{h_{c}}{2}}\rho_{c}dz_{1}$$

Thus, the material properties of the annular plate are

(12) $$E\left(z_{1}\right)=\{\begin{array}{ll} E_{f} & \frac{kh}{2}<z_{1}\leq \frac{h}{2}\\ E_{c}\left(z_{1}\right) & -\frac{kh}{2}\leq z_{1}\leq \frac{kh}{2}\\ E_{f} & -\frac{h}{2}\leq z_{1}<-\frac{kh}{2} \end{array}$$

(13) $$\mu \left(z_{1}\right)=\{\begin{array}{ll} \mu_{f} & \frac{kh}{2}<z_{1}\leq \frac{h}{2}\\ \mu_{c}\left(z_{1}\right) & -\frac{kh}{2}\leq z_{1}\leq \frac{kh}{2}\\ \mu_{f} & -\frac{h}{2}\leq z_{1}<-\frac{kh}{2} \end{array}$$

(14) $$\rho \left(z_{1}\right)=\{\begin{array}{ll} \rho_{f} & \frac{kh}{2}<z_{1}\leq \frac{h}{2}\\ \rho_{c}\left(z_{1}\right) & -\frac{kh}{2}\leq z_{1}\leq \frac{kh}{2}\\ \rho_{f} & -\frac{h}{2}\leq z_{1}<-\frac{kh}{2} \end{array}$$

where *k* = *h_c_*/*h* is the ratio of the core thickness to annular plate thickness; *E_f_*, *μ_f_* and *ρ_f_* are Young’s modulus, Poisson’s ratio and mass density of the face sheet, respectively.

### 2.3. Energy Functions

To obtain the equation of motion of the spinning sandwich annular plate, the energy method is applied.

The displacements of the annular plate (*r_x_*, *r_y_*, *r_z_*) are

(15) $$\left\{ \begin{array}{l} r_{x}=r\cos\theta \cos\left(\Omega t\right)-r\sin\theta \sin\left(\Omega t\right)-r\cos\theta \\ r_{y}=r\cos\theta \sin\left(\Omega t\right)+r\sin\theta \cos\left(\Omega t\right)-r\sin\theta \\ r_{z}=w\left(r,\theta \right) \end{array}\right.$$

in which *w* is the deflection displacement.

The velocities of the annular plate are

(16) $$\left\{ \begin{array}{l} v_{x}=-\Omega r\cos\theta \sin\left(\Omega t\right)-\Omega r\sin\theta \cos\left(\Omega t\right)\\ v_{y}=\Omega r\cos\theta \cos\left(\Omega t\right)-\Omega r\sin\theta \sin\left(\Omega t\right)\\ v_{z}=\dot{w}\left(r,\theta \right) \end{array}\right.$$

Thus, its kinetic energy can be obtained as

(17) $$\begin{array}{l} T=\frac{1}{2}\int_{V}\rho \left(v_{x}{}^{2}+v_{y}{}^{2}+v_{z}{}^{2}\right)dV\\ =\frac{\pi }{4}\Omega^{2}\int_{-\frac{h}{2}}^{\frac{h}{2}}\rho dz_{1}\left(R_{b}{}^{4}-R_{a}{}^{4}\right)+\frac{1}{2}\int_{-\frac{h}{2}}^{\frac{h}{2}}\rho dz_{1}\int_{R_{a}}^{R_{b}}\int_{0}^{2\pi }{\dot{w}}^{2}rdrd\theta \end{array}$$

Based on the Kirchhoff plate theory, the strain and displacement can be related by

(18) $$\left\{ \begin{array}{l} \varepsilon_{rr}=-z_{1}\frac{\partial^{2}w}{\partial r^{2}}\\ \varepsilon_{\theta \theta }=-z_{1}\left(\frac{1}{r}\frac{\partial w}{\partial r}+\frac{1}{r^{2}}\frac{\partial^{2}w}{\partial \theta^{2}}\right)\\ \varepsilon_{r\theta }=-2z_{1}\left(\frac{1}{r}\frac{\partial^{2}w}{\partial r\partial \theta }-\frac{1}{r^{2}}\frac{\partial w}{\partial \theta }\right) \end{array}\right.$$

According to the generalized Hooke law, one can obtain that

(19) $$\{\begin{array}{l} \sigma_{rr}=\frac{E}{1-\mu^{2}}\left(\varepsilon_{rr}+\mu \varepsilon_{\theta \theta }\right)\\ \sigma_{\theta \theta }=\frac{E}{1-\mu^{2}}\left(\varepsilon_{\theta \theta }+\mu \varepsilon_{rr}\right)\\ \sigma_{r\theta }=\frac{E}{2(1+\mu )}\varepsilon_{r\theta } \end{array}$$

Due to the deformation, the potential energy of the annular plate can be derived as

(20) $$\begin{array}{l} V_{1}=\frac{1}{2}\int_{0}^{2\pi }\int_{R_{a}}^{R_{b}}\int_{-\frac{h}{2}}^{\frac{h}{2}}\left[\sigma_{rr}\varepsilon_{rr}+\varepsilon_{\theta \theta }\sigma_{\theta \theta }+\varepsilon_{r\theta }\sigma_{r\theta }\right]rdz_{1}drd\theta \\ \;=\frac{1}{2}\int_{0}^{2\pi }\int_{R_{a}}^{R_{b}}\int_{-\frac{h}{2}}^{\frac{h}{2}}\frac{Ez_{1}{}^{2}}{\left(1-\mu^{2}\right)}\left\{\begin{array}{l} {\left(\frac{\partial^{2}w}{\partial r^{2}}+\frac{\partial w}{r\partial r}+\frac{\partial^{2}w}{r^{2}\partial \theta^{2}}\right)}^{2}+2\left(1-\mu \right){\left[\frac{\partial }{\partial r}\left(\frac{1}{r}\frac{\partial w}{\partial \theta }\right)\right]}^{2}\\ -2\left(1-\mu \right)\frac{\partial^{2}w}{\partial r^{2}}\left(\frac{1}{r}\frac{\partial w}{\partial r}+\frac{1}{r^{2}}\frac{\partial^{2}w}{\partial \theta^{2}}\right) \end{array}\right\}rdz_{1}drd\theta \end{array}$$

Because of the rotation effect, the plane strain can be given by

(21) $$\varepsilon_{rr}{}^{0}=\frac{\partial u^{0}}{\partial r},\;\varepsilon_{\theta \theta }{}^{0}=\frac{u^{0}}{r}$$

where *u*^0^ is the plane displacement.

On the basis of the generalized Hooke law, the plane stress is

(22) $$\left\{ \begin{array}{c} \sigma_{rr}{}^{0}=\frac{E}{1-\mu^{2}}\left(\varepsilon_{rr}{}^{0}+\mu \varepsilon_{\theta \theta }{}^{0}\right)=\frac{E}{1-\mu^{2}}\left(\frac{\partial u^{0}}{\partial r}+\mu \frac{u^{0}}{r}\right)\\ \sigma_{\theta \theta }{}^{0}=\frac{E}{1-\mu^{2}}\left(\varepsilon_{\theta \theta }{}^{0}+\mu \varepsilon_{rr}{}^{0}\right)=\frac{E}{1-\mu^{2}}\left(\frac{u^{0}}{r}+\mu \frac{\partial u^{0}}{\partial r}\right) \end{array}\right.$$

In terms of equilibrium condition and boundary conditions

(23) $$\frac{\partial \sigma_{rr}{}^{0}}{\partial r}+\frac{\sigma_{rr}{}^{0}-\sigma_{\theta \theta }{}^{0}}{r}+\rho \Omega^{2}r=0$$

(24) $${u^{0}|}_{r=R_{a}}=0,\;{\sigma_{rr}{}^{0}|}_{r=R_{b}}=0$$

the plane displacement *u*^0^ can be obtained as

(25) $$u^{0}=-\frac{1-\mu^{2}}{8E}\rho \Omega^{2}\left[r^{3}+\frac{\kappa_{1}r}{1+\mu }+\frac{\kappa_{2}}{\left(1-\mu \right)r}\right]$$

where *κ*_1_ and *κ*_2_ are given in the Appendix A.

Due to the rotation effect, the potential energy of the annular plate is

(26) $$\begin{array}{l} V_{2}=\frac{1}{2}\int_{0}^{2\pi }\int_{R_{a}}^{R_{b}}\int_{-\frac{h}{2}}^{\frac{h}{2}}\left[\sigma_{rr}{}^{0}\varepsilon_{rr}{}^{0}+\sigma_{\theta \theta }{}^{0}\varepsilon_{\theta \theta }{}^{0}+\sigma_{rr}{}^{0}{\left(\frac{\partial w}{\partial r}\right)}^{2}+\sigma_{\theta \theta }{}^{0}{\left(\frac{1}{r}\frac{\partial w}{\partial \theta }\right)}^{2}\right]rdz_{1}drd\theta \\ \;=\frac{1}{128}\rho^{2}\Omega^{4}\frac{1-\mu^{2}}{E}\int_{0}^{2\pi }\int_{R_{a}}^{R_{b}}\int_{-\frac{h}{2}}^{\frac{h}{2}}\left[\begin{array}{l} +\left(1+3\mu \right)r^{6}+\left(9+3\mu \right)r^{5}+3\kappa_{1}r^{3}+\kappa_{1}r^{4}\\ -\frac{3+\mu }{1-\mu }\kappa_{2}r+\frac{1+3\mu }{1-\mu }\kappa_{2}r^{2}+\frac{2}{1-\mu^{2}}\kappa_{1}\kappa_{2}\\ -\frac{2}{1-\mu^{2}}\kappa_{1}\kappa_{2}\frac{1}{r}-\frac{9+3\mu }{1+\mu }\kappa_{1}\kappa_{2}r^{3}\\ +r\frac{1+3\mu }{1+\mu }\kappa_{1}\kappa_{2}r^{4}+\frac{1}{1+\mu }\kappa_{1}{}^{2}r+\frac{1}{1+\mu }\kappa_{1}{}^{2}r^{2}\\ +\frac{1}{1-\mu }\kappa_{2}{}^{2}\frac{1}{r^{3}}+\frac{1}{1-\mu }\kappa_{2}{}^{2}\frac{1}{r^{2}} \end{array}\right]dz_{1}drd\theta \\ \;-\frac{1}{16}\rho \Omega^{2}\int_{0}^{2\pi }\int_{R_{a}}^{R_{b}}\int_{-\frac{h}{2}}^{\frac{h}{2}}\left\{\begin{array}{l} \left[\left(3+\mu \right)r^{3}+\kappa_{1}r-\kappa_{2}\frac{1}{r}\right]{\left(\frac{\partial w}{\partial r}\right)}^{2}\\ +\left[\left(1+3\mu \right)r+\kappa_{1}\frac{1}{r}+\kappa_{2}\frac{1}{r^{3}}\right]{\left(\frac{\partial w}{\partial \theta }\right)}^{2} \end{array}\right\}dz_{1}drd\theta \end{array}$$

Finally, the total potential energy of the annular plate is

(27) $$V=V_{1}+V_{2}$$

### 2.4. Equations of Motion

The assumed modes method is employed in this paper. The displacement of the annular plate is assumed as

(28) $$w\left(r,\theta ,t\right)=\cos\theta \Phi \left(r\right)P{\left(t\right)}^{T}$$

in which **P**(*t*) is the generalized coordinate vector in the form of

(29) $$P\left(t\right)=\left[\begin{array}{cccc} p_{1}\left(t\right) & p_{2}\left(t\right) & \cdots & p_{n}\left(t\right) \end{array}\right]$$

and **Φ**(*r*) is the mode function vector, expressed as

(30) $$\Phi \left(r\right)=\left[\begin{array}{cccc} R_{1}\left(r\right) & R_{2}\left(r\right) & \cdots & R_{n}\left(r\right) \end{array}\right]$$

where *n* is the mode number.

The mode function *R_n_*(*r*) can be given by

(31) $$R_{n}\left(r\right)=A_{n}J_{1}\left(\beta_{n}r/R_{b}\right)+B_{n}N_{1}\left(\beta_{n}r/R_{b}\right)+C_{n}I_{1}\left(\beta_{n}r/R_{b}\right)+D_{n}K_{1}\left(\beta_{n}r/R_{b}\right)$$

in which *J*_1_ and *I*_1_ are first kind Bessel function and the modified one, respectively; *N*_1_ and *K*_1_ are second kind Bessel function and the modified one, respectively; *A_n_*, *B_n_*, *C_n_*, *D_n_* and *β_n_* can be determined by the boundary conditions of the annular plate.

According to the Lagrange equation

(32) $$\frac{d}{dt}\left(\frac{\partial L}{\partial {\dot{q}}_{i}}\right)-\frac{\partial L}{\partial q_{i}}=0,\;L=T-V$$

the equations of motion of the spinning annular plate can be derived as

(33) $$M\ddot{q}\left(t\right)+Kq\left(t\right)=0$$

where **M** and **K** are given in the Appendix A.

Setting

(34) $$q\left(t\right)=\psi e^{i\omega t},i=\sqrt{-1}$$

gives the eigenvalue equation

(35) $$\left(K-\omega^{2}M\right)\psi =0$$

where vector **ψ** is composed of unknown constants *A_n_*, *B_n_*, *C_n_*, *D_n_* (*n* = 1, …). The natural frequency *ω* can be obtained by solving the eigenvalue problem from Equation (35).

## 3. Results and Discussions

In this part, the effects of material parameters on the free vibration behaviors of the spinning annular plate with porous core reinforced by GPLs are examined in detailed. Unless otherwise stated, the structural and material parameters [34] are given in Table 1. In addition, porosity distribution pattern X*_P_* and GPL distribution pattern X*_G_* are taken as an example in the subsequent analysis.

### 3.1. Convergence and Comparison Study

Before parametric analysis, the convergence and comparison analysis have to be conducted first. Table 2 lists the variations of the first four natural frequencies with mode number by theoretical method (MATLAB), which shows that convergent frequencies can be obtained at *n* = 6.

**Figure 4:**
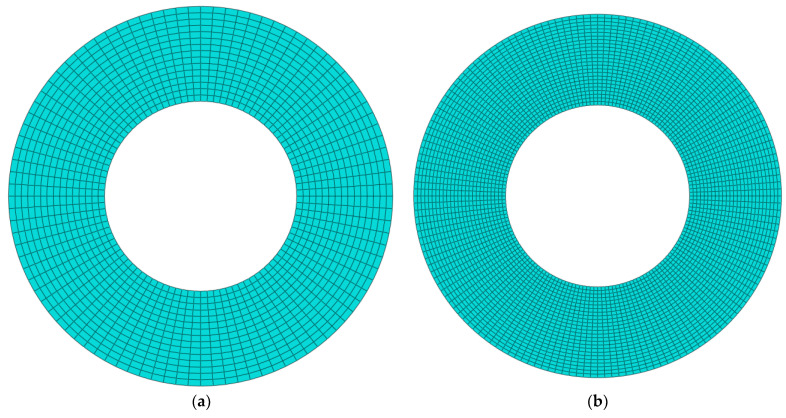

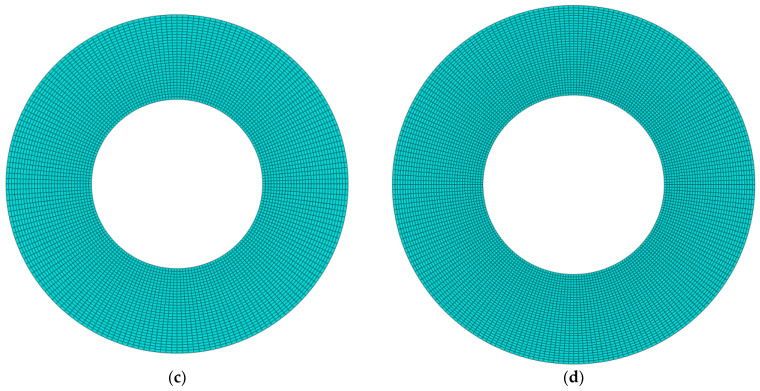
Mesh graph of different mesh element numbers. (**a**) *N_e_* = 1440 (**b**) *N_e_* = 4000, (**c**) *N_e_* = 5760 (**d**) *N_e_* = 7840.

The finite element method is used to validate the present analysis by using commercial software ABAQUS. The functionally graded material core is divided into ten layers and the material properties of each layer are calculated by the equations in Section 2.2. The annular plate is clamped at the inner edge, free at its outer edge, and is discretized by 4-node doubly curved general-purpose shell elements with 6 degrees of freedom. To examine the convergence of the finite element analysis, Table 3 gives the first four natural frequencies at *Ω* = 500 rad/s with different total numbers of elements *N*_e_ = (1440, 4000, 5760, 7840) and nodes (1536, 4160, 5952, 8064). Figure 4 displays the corresponding mesh graphs. It is clear that the free vibration results come to be converged at element number *N_e_* = 7840.

**Figure 5:**
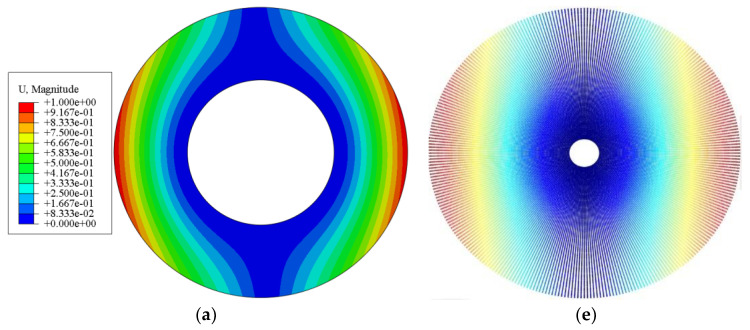

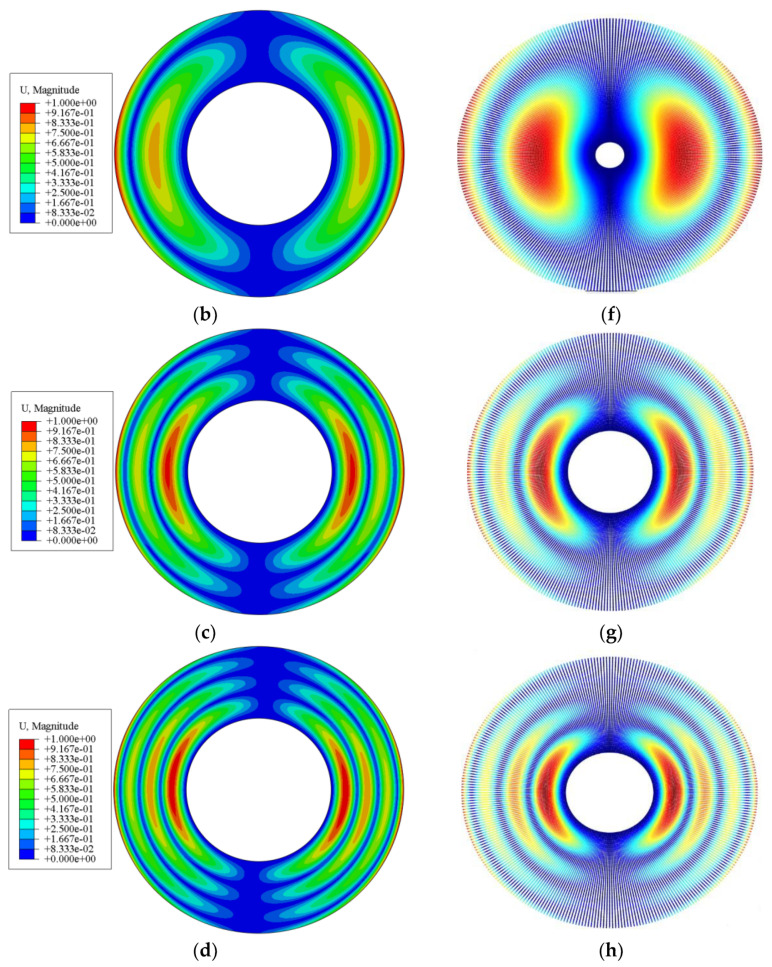
Vibration modes of the spinning annular plate: (**a**–**d**) are the first four vibration modes obtained by ABAQUS; (**e**–**h**) are the first four vibration modes obtained by MATLAB.

Table 4 and Figure 5 give the comparison of first four natural frequencies and vibration modes by theoretical method (MATLAB) and finite element (ABAQUS) method at *Ω* = 500 rad/s, respectively. It is obvious that the frequencies and vibration modes are in agreement, which shows that the present analysis is accurate.

In addition, the theoretical results are also compared with the experimental results [35] in Table 5, where the parameters are given in Table 6. One can see that the theoretical calculation results are in good agreement with the experimental results, which tells that the present analysis is accurate.

### 3.2. Parametric Analysis

In this section, both the graphic form and tabular form are utilized to conduct the parametric analysis on the free vibration results of the spinning FG annular plate with porous core reinforced by GPLs.

**Figure 6:**
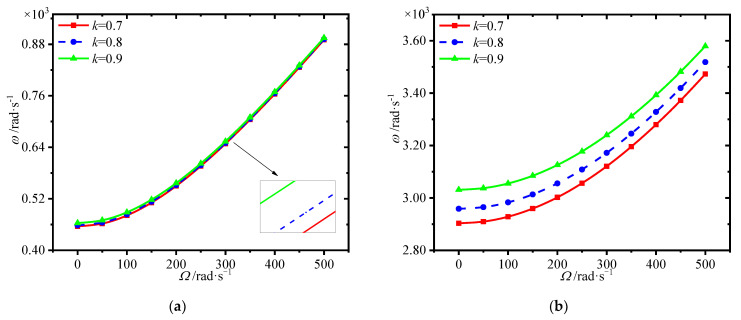

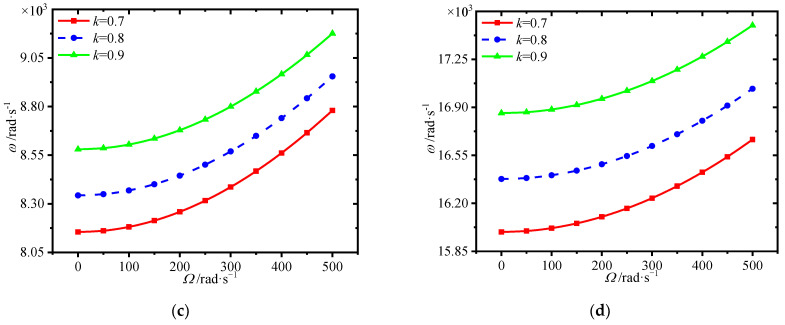
Variations of first four natural frequencies (rad/s) with spinning speed for different ratio of the core thickness to annular plate thickness. (**a**) first frequency (**b**) second frequency, (**c**) third frequency (**d**) fourth frequency.

Figure 6 depicts the variations of first four natural frequencies of the annular plate with spinning speed for different ratios of the core thickness to annular plate thickness. A considerable rise in the frequencies is observed as the spinning speed increases. In addition, the larger ratio of the core thickness to annular plate thickness leads to greater frequencies. It indicates that thinner faces could be adopted in the present sandwich structure to achieve better mechanical performance.

**Figure 7:**
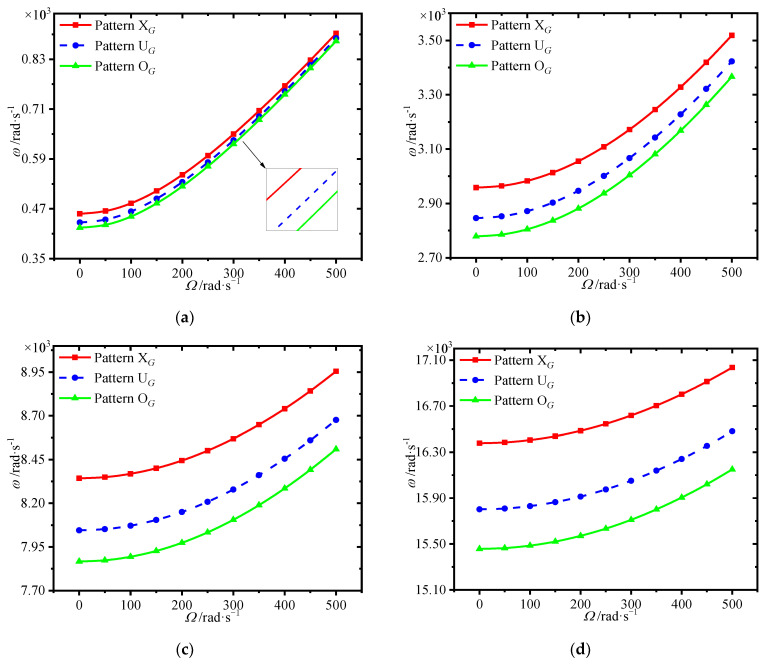
Variations of first four natural frequencies (rad/s) with spinning speed for different GPL distributions. (**a**) first frequency (**b**) second frequency, (**c**) third frequency (**d**) fourth frequency.

Figure 7 plots the variations of first four natural frequencies of the annular plate with spinning speed for different GPL distributions. It is seen that the GPL distribution pattern X*_G_* provides highest frequencies, while pattern O*_G_* has the worst enhancement effect. This implies that dispersing more GPLs around the surfaces of the core could give a hand to enhance the structural stiffness.

**Figure 8:**
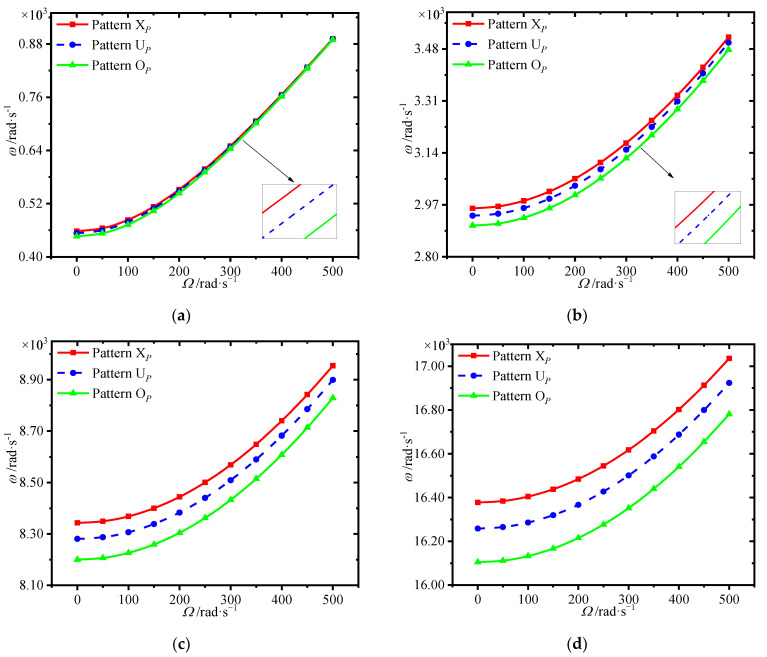
Variations of first four natural frequencies (rad/s) with spinning speed for different porosity distributions. (**a**) first frequency (**b**) second frequency, (**c**) third frequency (**d**) fourth frequency.

The variations of first four natural frequencies of the annular plate with spinning speed for different porosity distributions are presented in Figure 8. Results show that porosity distribution pattern X*_P_* affords greatest frequencies, while the pattern O*_P_* gives the smallest one. It is noted that setting more pores around the surfaces of the core is effective to obtain great mechanical performance.

Since the variations of natural frequencies with spinning speed are similar, only two typical spinning speeds, 0 rad/s and 500 rad/s, are adopted in the following analysis.

**Figure 9:**
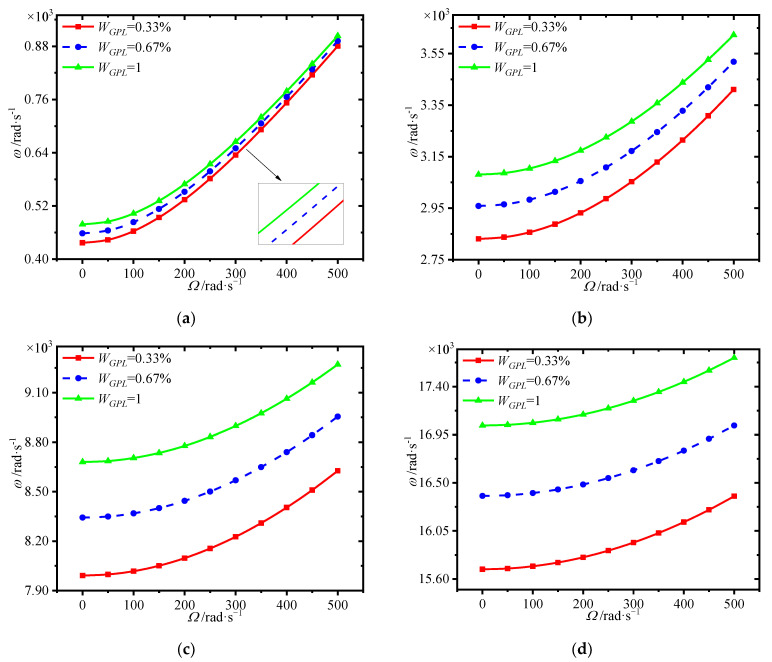
Variations of first four natural frequencies (rad/s) with GPL weight fraction for different spinning speeds. (**a**) first frequency (**b**) second frequency, (**c**) third frequency (**d**) fourth frequency.

Figure 9 shows the variations of first four natural frequencies of the annular plate with GPL weight fraction at different spinning speeds. One can see that the frequencies increase markedly with the GPL weight fraction. It is worth noting that adding more GPLs into the core plays a very important role in obtaining greater enhancement.

**Figure 10:**
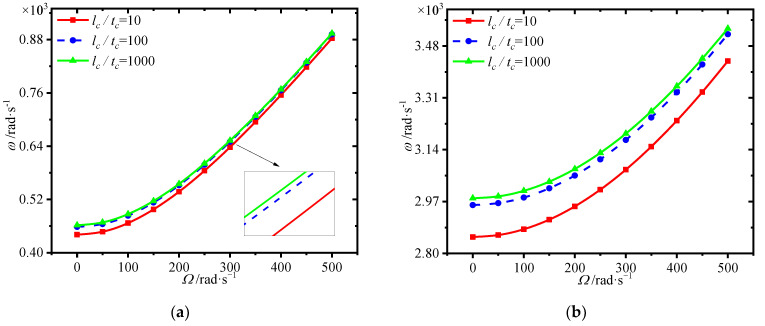

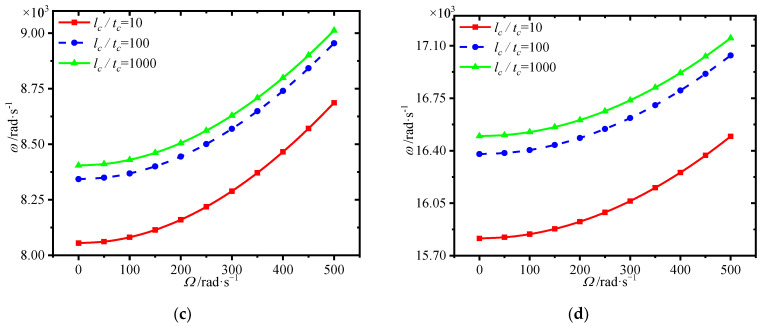
Variations of first four natural frequencies (rad/s) with GPL length-to-thickness ratio for different spinning speeds. (**a**) first frequency (**b**) second frequency, (**c**) third frequency (**d**) fourth frequency.

Figure 10 lists the variations of first four natural frequencies of the annular plate with GPL length-to-thickness ratio at different spinning speeds. We can see that the frequencies rise dramatically with the GPL length-to-thickness ratio. For the same content of GPLs, larger GPL length-to-thickness ratio means a thinner GPL. It can be seen that better enhancement effect occurs when thinner GPLs are added into the core.

**Figure 11:**
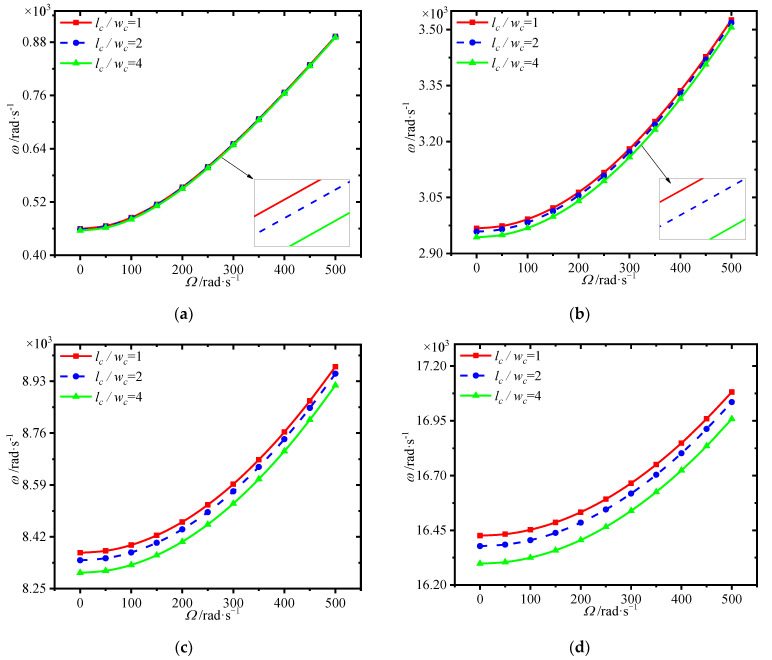
Variations of first four natural frequencies (rad/s) with GPL length-to-width ratio for different spinning speeds. (**a**) first frequency (**b**) second frequency, (**c**) third frequency (**d**) fourth frequency.

Figure 11 gives the variations of first four natural frequencies of the annular plate with GPL length-to-width ratio at different spinning speeds, where GPL length remains constant. It is seen that the frequencies are reduced with a rise in GPL length-to-width ratios. Here it should be noted that a smaller GPL length-to-width ratio means each GPL with larger surface area, which can lead to better load transfer capacity.

**Figure 12:**
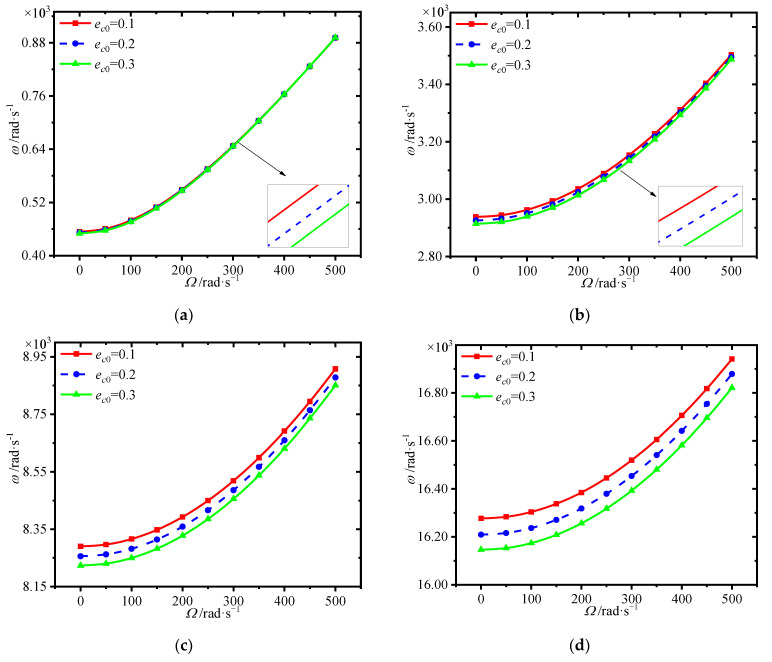
Variations of first four natural frequencies (rad/s) with porosity coefficient for different spinning speeds. (**a**) first frequency (**b**) second frequency, (**c**) third frequency (**d**) fourth frequency.

The variations of first four natural frequencies of the annular plate with porosity coefficient at different spinning speeds is presented in Figure 12. One can see that the frequencies decrease in general with the increase of porosity coefficient. Although the larger porosity coefficient can result in light weight, it weakens the structural stiffness.

## 4. Conclusions

This paper concerned with the free vibration behavior of a spinning FG annular plate with porous core reinforced by GPLs. Based on the Kirchhoff plate theory, the equations of motion are obtained by employing the Lagrange equation method. The model and vibration analysis are verified by adopting the FE method. Several interesting results could be noted as follows.

(1) thinner faces could be adopted in the present sandwich structure to achieve better mechanical performance.
(2) setting more pores and GPLs around the surfaces of the core is effective in enhancing the structural stiffness.
(3) adding a few GPLs into the core plays a very important role in obtaining greater enhancement.
(4) better enhance effect occurs when thinner GPLs with larger surface areas are applied to be added into the core.
(5) larger porosity coefficient can result in light weight and weaken the structural stiffness.

## Figures and Tables

**Table 1:** The structural and material parameters.

Parameters	Value
*R_a_*	0.5 m
*R_b_*	1 m
*h*	0.02 m
*k*	0.8
*E_GPL_*	1010 GPa
*ρ_GPL_*	1062.5 kg/m^3^
*μ_GPL_*	0.186
*E_M_*	68.3 GPa
*E_f_*	68.3 GPa
*ρ_M_*	2689.8 kg/m^3^
*ρ_f_*	2689.8 kg/m^3^
*μ_M_*	0.34
*μ_f_*	0.34
*W_GPL_*	0.67%
*l_c_*/*t_c_*	100
*L_c_*/*w_c_*	2
*e_c0_*	0.1

**Table 2:** First four natural frequencies (rad/s) of the spinning annular plate with different mode numbers (Ω = 500 rad/s).

Frequency (rad/s)	*n* = 4	*n* = 5	*n* = 6	*n* = 7
First	892.13	892.06	892.02	892.01
Second	3518.88	3518.56	3518.44	3518.36
Third	8955.60	8954.89	8954.70	8954.58
Fourth	17,039.40	17,035.83	17,035.43	17,035.32

**Table 3:** First four natural frequencies (rad/s) of the spinning annular plate with different element numbers by finite element (FE) method (*Ω* = 500 rad/s).

Frequency (Hz)	*N_e_* = 1440	*N_e_* = 4000	*N_e_* = 5760	*N_e_* = 7840
First	140.96	140.82	140.80	140.79
Second	555.15	551.85	551.29	550.95
Third	1421.00	1395.70	1391.50	1388.90
Fourth	2728.30	2630.20	2613.90	2604.10

**Table 4:** Comparison of first four natural frequencies of the spinning annular plate by theory method and finite element (FE) method (*Ω* = 500 rad/s).

Frequency	Present (Hz)	FE (Hz)	Error
First	141.97	140.79	0.84%
Second	559.98	550.95	1.64%
Third	1425.18	1388.90	2.61%
Fourth	2711.27	2604.10	4.12%

**Table 5:** Comparison of first two natural frequencies of a spinning annular plate between theory method and experiment method [35] (*Ω* = 0 rad/s).

Frequency	Present (Hz)	Experiment (Hz)	Error
First	38.95	37.19 ± 0.29	4.73%
Second	265.35	262.38 ± 1.42	1.01%

**Table 6:** The structural and material parameters in the literature [35].

Parameter	Value
*R_a_*	178 mm
*R_b_*	53.35 mm
*h*	0.775 mm
*E*	200 GPa
*ρ*	7840 kg/m^3^
*μ*	0.3

## Data Availability

Data sharing is not applicable to this article.

## Appendix A

(A1)
$$\left\{ \begin{array}{l} \kappa_{1}=-\frac{\left(1+\mu \right)\left(3+\mu \right)R_{b}{}^{4}+\left(1-\mu^{2}\right)R_{a}{}^{4}}{\left(1+\mu \right)R_{b}{}^{2}+\left(1-\mu \right)R_{a}{}^{2}}\\ \kappa_{2}=R_{b}{}^{2}R_{a}{}^{2}\frac{\left(1-\mu \right)\left(3+\mu \right)R_{b}{}^{2}-\left(1-\mu^{2}\right)R_{a}{}^{2}}{\left(1+\mu \right)R_{b}{}^{2}+\left(1-\mu \right)R_{a}{}^{2}} \end{array}\right.$$

(A2)
$$M=\pi \int_{-\frac{h}{2}}^{\frac{h}{2}}\rho dz_{1}\int_{R_{a}}^{R_{b}}\Phi^{T}\Phi rdr$$

(A3)
$$K=\pi \int_{a}^{b}\int_{-\frac{h}{2}}^{\frac{h}{2}}\left\{\begin{array}{l} \frac{Ez_{1}{}^{2}}{1-\mu^{2}}\;r\;{\Phi^{\prime \prime }}^{T}\;\Phi^{\prime \prime }+\frac{Ez_{1}{}^{2}}{1-\mu^{2}}\mu \left({\Phi^{\prime \prime }}^{T}\;\Phi^{\prime }+\;{\Phi^{\prime }}^{T}\Phi^{\prime \prime }\;\right)+\frac{Ez_{1}{}^{2}}{1-\mu^{2}}\frac{\left(3-2\mu \right)}{r}{\Phi^{\prime }}^{T}\;\Phi^{\prime }\\ +\frac{\rho \Omega^{2}}{8}[-\left(3+\mu \right)r^{3}-\kappa_{1}r+\frac{\kappa_{2}}{r}]{\Phi^{\prime }}^{T}\;\Phi^{\prime }+\frac{\rho \Omega^{2}}{8}[-\left(1+3\mu \right)r-\frac{\kappa_{1}}{r}-\frac{\kappa_{2}}{r^{3}}]\Phi^{T}\Phi \\ +\frac{Ez_{1}{}^{2}}{1-\mu^{2}}\frac{\left(3-2\mu \right)}{r^{3}}\Phi^{T}\Phi -\frac{Ez_{1}{}^{2}}{1-\mu^{2}}\frac{\;\mu }{r}\left({\Phi^{\prime \prime }}^{T}\;\Phi +\Phi^{T}\;\Phi^{\prime \prime }\right)\\ -\frac{Ez_{1}{}^{2}}{1-\mu^{2}}\frac{\left(3-2\mu \right)}{r^{2}}\left({\Phi^{\prime }}^{T}\;\Phi +\;\Phi^{T}\Phi^{\prime }\;\right) \end{array}\right\}dz_{1}dr$$
